# A national intercalated medical student research program – student perceptions, satisfaction, and factors associated with pursuing a PhD

**DOI:** 10.1080/10872981.2022.2122105

**Published:** 2022-09-07

**Authors:** Marie Søfteland Sandvei, Geir Wenberg Jacobsen, Marianne Heldal Stien, Helge Ræder, Ludvig Andre Munthe, Vegard Skogen

**Affiliations:** a Department of Public Health and Nursing, Norwegian University of Science and Technology - NTNU, Trondheim, Norway; b The Cancer Clinic, St Olavs Hospital, Trondheim University Hospital, Trondheim, Norway; c Faculty of Medicine, University of Bergen, Bergen, Norway; d Department of Clinical Science, University of Bergen, Bergen, Norway; e KG Jebsen Centre for Bcell Malignancies, Faculty of Medicine, University of Oslo, Oslo, Norway; f Faculty of Health Sciences, UiT Arctic University of Norway, Tromsø, Norway; g Department of Infectious Diseases, Division of Internal Medicine, University Hospital of North Norway, Tromsø, Norway

**Keywords:** Medical education, undergraduate research, medical student, satisfaction, supervision

## Abstract

**Background:**

To counteract a decreasing number of physician-scientists, a national intercalated Medical Student Research Programme (MSRP) was launched in Norway in 2002. We aimed to assess whether the students’ favourable perceptions and satisfaction with the program had prevailed since the inception in 2002 and until 2015, and to identify factors associated with pursuing a PhD.

**Methods:**

The study was an incorporation of data from two previous national evaluations of the MSRP performed in 2007 and 2015. We used electronic questionnaires to explore demographic characteristics, area and type of research, student satisfaction, and future scientific goals. In 2007, questionnaires were sent to all 208 students, and 183 (88%) replied. In 2015, the corresponding numbers were 279, and 240 (86%). Categorical data were analysed using either Kruskal-Wallis or Pearson’s chi square test. Differences between sample means were assessed with Student`s t-test while logistic regression was used to test associations between selected covariates and the students’ ambitions to pursue a PhD degree.

**Results:**

Overall, the student satisfaction was 79%. However, more students in 2015 received less regular and less supervision time and expressed a need for more of it. Seventy-seven per cent expressed an ambition to pursue a PhD. Students were more likely to have a PhD ambition if they were satisfied with the program, had a supervisor with high expectations for them, or had already published some of their results. At both time points, students (86% vs. 89%) responded that the MSRP had a positive impact on their regular curriculum achievements.

**Conclusions:**

The high degree of satisfaction with the national MSRP among undergraduate students has prevailed since the inception in 2002. By far, the program has also met its goal to increase the number of aspiring physician-scientists. However, to maintain that goal over time, adequate and personal supervision is a prerequisite.

## Introduction

Over the years various initiatives have aimed to encourage undergraduate medical students and candidates to pursue a scientific and academic track career [1–6]. Descriptive studies have employed both qualitative, quantitative, and mixed methods design. Some of the early papers discussed a potential impact from local undergraduate intercalated research programs on later clinical understanding and practice [1,3]. Others argued in favour of a more mandatory approach, that takes future recruitment of medical academics, an extended role of evidence-based medicine as well as the interpretation of meta-analyses into account [2,4,5]. An important study of enablers and constraints of motivation to conduct undergraduate research underscored the importance of meeting the students’ needs for autonomy, competence, and relatedness [6].

A series of recent papers by a Dutch group have documented the potential to motivate undergraduate medical research [7–9]. Like others, they are concerned with a future shortage of physician-scientists and focus on motivation of as early as first-year students. That includes even participation in active conduct of research [7]. Moreover, in a countrywide study among graduates from all eight Dutch universities, they showed that students who had published before graduation, were almost twice as likely to publish later and more during their career than their peers [8]. Thirdly, the same group of authors reported the results of a qualitative study where medical students demonstrated insight into motivating as well as demotivating factors to conduct research [9]. Similar outcomes were reported from a cross-sectional study by a group of authors at the American University of Beirut, Lebanon [10]. A quite recent paper by van Rooij et al. includes an impressive overview of the literature of factors that influence what they call ‘the challenges of the doctoral journey’ [11]. High on that list are concerns about the extensive dropout (attrition) rates and progress delay among PhD students, both of which have institutional as well as personal economic costs. Another relevant concern is that student dissatisfaction and delay may result in negative mental health issues of various kinds. From the wealth of the literature, the authors have identified three domains or categories that predict a positive outcome of this ‘doctoral journey’ in terms of student satisfaction and retention. These domains are characterized by the ‘academic culture’ of the institution, the student-supervisor interaction, and the student’s characteristics, i.e., personality and motivation. The three core factors are the student’s academic competence and motivation, a favourable relation with the supervisor and integration in his/her research unit, as well as a sufficient portion of student autonomy to ascertain ‘ownership’ of the project [11].

An obvious lack in recruitment to academic medicine in Norway also caused concern [12,13], and action was taken jointly by the four Norwegian Faculties of Medicine, the Research Council of Norway, and the Norwegian Department of Education and Science. As a result, a national Medical Student Research Program (MSRP) was conceived and funded in 2002. It was organized in concert and launched simultaneously at all four faculties. Each medical faculty has been granted earmarked funds from government authorities based on the agreed annual quota of new students. Mostly, 10% of each incoming medical student cohort is admitted through an annual application procedure. The MSRP is a two-year course (120 European Credit Transfer and Accumulation System (ECTS)), where one full academic year is added to the regular six-year program. Further, one more year is integrated as extra time dedicated to scientific work during weekends, holidays, and summer breaks. The required educational component of the regular doctoral programme (PhD) consists of 30 credits (ECTS) and is part of the MSRPs curriculum of 120 credits. Thus, the total duration required for the study of medicine including the MSRP is increased by one year to a total of seven years [14]. Within these frames, each faculty is responsible for the admission process, planning, and conduct of the program.

Since its inception, two evaluation surveys of the program have been conducted (Evaluation 1 (E1), 2007 and Evaluation 2 (E2), 2015) [14,15]. The report from the first survey provided details about the format and content of the MSRP as well as early experiences among the students and their supervisors. It showed that the MSRP had led to an increase in the recruitment of graduated physicians to medical research in Norway [14]. Based on data from the second survey, we have previously reported that up to eight years after graduation as MDs, ten times more MSRP candidates had completed their PhD compared to a representative sample of their non-MSRP colleagues [15].

The students` perception and satisfaction with the program were important elements of both evaluations. Here, we compare such outcomes between the first [14] and second [15] survey (E1 and E2). The aim of our study was to assess whether the students’ perception and satisfaction were sustained since the Norwegian intercalated MSRP was launched in 2002 and until 2015, and to identify factors associated with student ambitions to pursue a PhD.

## Materials and methods

### Study population

The Research Council of Norway – as the funding agency – required comprehensive evaluations of the MSRP from the Medical faculties. To meet this requirement, two electronic questionnaire surveys were performed among MSRP students, in 2007 and 2015 [14,15]. Due to the uniform national framework of the program, i.e., common goals, uniform organisation and funding, and date of inception, we decided to label the students’ home institutions as University 1 to 4.

### Questionnaire and data collection

The 2007 questionnaire was designed by a working group appointed by the four medical deans [14], and was distributed by email to MSRP students with an information and invitation to take part in the national evaluation. The same procedure was followed in 2015 and questionnaires used in 2007 and 2015 were almost identical [14,15]. The questionnaires had quantitative and categorical answer options. Still, in 2007 respondents had the opportunity to provide more extensive comments and answers to some questions. Also, there were specific questions for supervisors, an option that was not part of the 2015 questionnaire. The latter version, however, asked if candidates had considered resigning from the MSRP and if so, the reason for these considerations [15]. Overall, the 2007 and 2015 questionnaires had 39 and 26 items, respectively. They explored demographic characteristics such as the students’ gender, year of birth, ethnic background, which medical school, and year of entry as a student. Further, questions included classification and details of their research project, as well as their personal aims for choosing a student research track. Other questions explored the amount and frequency of supervision the students received and their satisfaction with their supervisor. Also, we asked about scientific output in terms of publications and manuscripts, dissemination of research results at national/international conferences, or any organised field studies abroad. Finally, we included questions about satisfaction with the service from their local research program administration, ambitions about and admission to subsequent PhD projects, whether the MSRP had a positive impact on their regular medical curriculum, and a global satisfaction with the program.

The 2007 electronic questionnaire employed the Questback program (Questback AS, Oslo, Norway) whereas the 2015 questionnaire used the program SurveyXact (Rambøl Management Consulting, Oslo, Norway). Data in E1 were collected between 15 January and 1 February 2007 and E2 from 19 January to 19 February 2015.

### Data analyses

To avoid too many categories with few answers in each, we merged items with five possible answer categories into three. Thus, in questions regarding satisfaction, the categories ‘very dissatisfied’ and ‘somewhat dissatisfied’ were classified as ‘dissatisfied’, while ‘somewhat satisfied’ and ‘very satisfied’ were termed ‘satisfied’. Correspondingly, ‘very low expectations’ and ‘low expectations’ were combined into ‘low expectations’, while ‘high expectations’ and ‘very high expectations’ were combined into ‘high expectations’. For the logistic regression analyses, the categories ‘no’ and ‘unsure’ were combined, thus creating a dichotomous variable of the students’ ambition to pursue a PhD as ‘No/Unsure’ vs. ‘Yes’.

Categorical ordinal and non-ordinal data were analysed using the Kruskal-Wallis and Pearson’s chi-square tests, respectively. Differences in mean age were tested using Student’s t-test. Logistic regression was used to explore associations between selected covariates and the students’ ambitions to pursue a PhD degree. A p-value < 0.05 and 95% confidence limits that excluded the null value of 1 was used as threshold to indicate statistical significance. All analyses were performed in Stata, Version 15 (StataCorp. 2017, College Station, TX, USA).

## Results

In E1 (2007), 183 out of 208 (88%) MSRP students completed and returned the evaluation questionnaire. The corresponding numbers in E2 (2015) were 240 out of 279 (86%) students. Table 1 shows characteristics of the participants. Most students were in their mid-20ies, but the E2 participants were slightly older than E1 ones (25.4 vs. 24.7 years, p = 0.01). The proportion of women was 43% in E1 and 48% in E2 (p = 0.38). In E1, most students were engaged in laboratory research, whereas the research areas were more diverse in E2. Almost half of the students (43%) had published a scientific paper or were about to do so. In E2, 45 (19%) students confirmed that they had considered to resign from the MSRP. The most prevalent reasons were reported as ‘personal’ (33%), loss of interest in their project (31%), and a non-optimal relationship with their supervisor (24%).

**Table 1.** Background characteristics of the first (E1) and second (E2) evaluation of the Norwegian medical student research program (MSRP) cohort.

	Evaluation Period 1– 2002-6	Evaluation Period 2–2007-14	Total
	N (%)	N (%)	N (%)
All participants	183	240	423
Gender			
Female	79 (43)	114 (48)	193 (46)
Male	104 (57)	126 (53)	230 (54)
Age mean, years (SD)	24.7 (2.8^a^)	25.4 (3.0^a^)	25.1 (2.9^a^)
Age categories, years			
20–23	63 (35)	67 (28)	130 (31)
24–26	77 (43)	104 (43)	181 (43)
27–29	32 (18)	46 (19)	78 (19)
30 and above	9 (5)	23 (10)	32 (8)
Area of research			
Laboratory medicine	109 (60)	115 (48)	224 (53)
Epidemiology	22 (12)	48 (20)	70 (17)
Clinical science	41 (22)	60 (25)	101 (24)
Combination	5 (3)	3 (1)	8 (2)
Other	6 (3)	11 (5)	17 (4)
Missing	0 (0)	3 (1)	3 (1)
University			
1	18	33	51
2	46	51	97
3	58	82	140
4	61	74	135
No. of Medical School Semesters before MSRP			
1–2	45 (25)	3 (1)	48 (11)
3–4	102 (56)	134 (56)	236 (56)
5–6	27 (15)	81 (34)	108 (26)
More than 6	8 (4)	20 (8)	28 (7)
Missing	1 (1)	2 (1)	3 (1)
No. of MSRP semesters before study participation			
Less than 2	40 (22)	44 (19)	84 (20)
2–3	43 (24)	59 (25)	102 (24)
4–5	52 (29)	63 (26)	115 (27)
6 or more	47 (26)	71 (30)	118 (28)
Missing	1 (1)	3 (1)	4 (1)
Ambitions to pursue a PhD			
No	4 (2)	7 (3)	11 (3)
Unsure	33 (18)	40 (17)	73 (17)
Yes	145 (79)	179 (75)	324 (77)
Missing	1 (1)	14 (6)	15 (4)
Publications			
No	92 (51)	146 (61)	238 (56)
To be submitted/Under review^b^	55 (30)	22 (9)	77 (18)
Yes	35 (19)	72 (30)	107 (25)
Missing	1 (1)	0 (0)	1 (0)
Considered resigning from the MSRP (n = 240)			
No	-	193 (80)	-
Yes	-	45 (19)	-
Missing	-	2 (1)	-
Reason for considered quitting (n = 45)			
Personal reasons	-	15 (33)	-
Lost interest in the project	-	14 (31)	-
Problematic relationship with supervisor	-	11 (24)	-
Did not ‘find my place’ in the research group	-	4 (9)	-
Missing		1 (2)	-

^a^Standard deviation (s.d.)

^b^The proposed answer differed between Evaluation Period 1: ‘I am about to publish a paper’ and Evaluation Period 2: ”Under review”

Four out of five students (79%) were satisfied with the MSRP in general (Table 2), and male students were more satisfied than females (84% vs. 74%, p = 0.02). The data suggested that students may have been more satisfied in E1 than in E2 (p = 0.055). However, there were no differences between the universities or any area of research.

**Table 2.** Students’ satisfaction with the Norwegian medical student research program (MSRP).

	Dissatisfied, n (%)	Neither dissatisfied nor satisfied, n (%)	Satisfied, n (%)	Total	Kruskal-Wallis test, p-value
All participants	40 (10)	44 (11)	325 (79)	409^a^	
Gender					0.02
Female	19 (10)	29 (16)	136 (74)	184	
Male	21 (9)	15 (7)	189 (84)	225	
Area of research^b^					0.88
Laboratory medicine	21 (10)	21 (10)	177 (81)	219	
Epidemiology	4 (6)	11 (17)	51 (77)	66	
Clinical	13 (13)	10 (10)	76 (77)	99	
Combination	1 (13)	0	7 (88)	8	
Other	1 (6)	2 (12)	14 (82)	17	
Evaluation Period					0.055
1: 2002–2006	13 (7)	17 (9)	153 (84)	183	
2: 2007–2014	27 (12)	27 (12)	172 (76)	226	
Students’ Home University					0.18
University 1	1 (2)	8 (16)	41 (82)	50	
University 2	6 (6)	7 (7)	82 (86)	95	
University 3	14 (10)	19 (14)	104 (76)	137	
University 4	19 (15)	10 (8)	98 (77)	127	

^a^Missing information for 14 participants

^b^Four possible answers; ‘public health/epidemiology’, ‘clinical/patient oriented’, ‘laboratory’, and ‘other, please specify’. The responses in the ‘other’ category were categorized as ‘combination’ if it included a combination of laboratory and either clinical or epidemiological research, and the ‘other’ category otherwise.

A clear majority of students (72%) were highly satisfied with the supervision they received (Table 3). However, students in E2 apparently received less regular and less frequent supervision and reported that they needed more. As perceived by the students, it seemed that the expectations among supervisors were higher for female than male students (74% vs 61% with ‘high expectations’, p = 0.004; data not shown). Yet, there were no differences in terms of area of research, evaluation period, or university. Three out of four students (75%) were satisfied with their own research efforts, but with no clear differences by gender, area of research, evaluation period, or university (data not shown). Similarly, a majority of students (68%) were satisfied with the MSRP administration at their university with no differences by gender, area of research, evaluation period, or university (data not shown). A clear majority of students reported that the MSRP had a positive impact on their other academic achievements (i.e., medical studies) to some (38%) or a large/very large degree (51%).

**Table 3.** Students’ satisfaction with their supervision in the Norwegian medical student research program (MSRP).

	Evaluation Period 1–2002-6	Evaluation Period 2–2007-14	Total	Kruskal-Wallis test/ Pearson’s Chi square test, p-value^a,b^
	N (%)	N (%)	N (%)	
Degree of satisfaction with the supervision				0.14^a^
To a small degree	4 (2)	19 (8)	23 (5)	
To some degree	40 (22)	51 (21)	91 (22)	
To a high degree	139 (76)	167 (70)	306 (72)	
Missing	0	3 (1)	3 (1)	
Regularity of supervision				0.013^a^
No supervision	1 (1)	5 (2)	6 (1)	
Sporadic	57 (31)	88 (37)	145 (34)	
Regular, but more seldom than every two weeks	26 (14)	36 (15)	62 (15)	
About every two weeks	34 (19)	54 (23)	88 (21)	
At least once every week	65 (36)	54 (23)	119 (28)	
Missing	0	3 (1)	3 (1)	
No. of hours of supervision over the last academic year				0.06^a^
Less than 20	56 (31)	91 (38)	147 (35)	
20–40	72 (39)	87 (36)	159 (38)	
41–80	36 (20)	46 (19)	82 (19)	
More than 80	19 (10)	13 (5)	32 (8)	
Missing	0	3 (1)	3 (1)	
Perceived need for more supervision				0.014^b^
No	125 (68)	128 (53)	253 (60)	
Do not know	23 (13)	41 (17)	64 (15)	
Yes	35 (19)	67 (28)	102 (24)	
Missing	0	4 (2)	4 (1)	

^a^Categorical ordinal data were analysed using the Kruskal-Wallis test.

^b^Categorical non-ordinal data were analysed using the Pearson’s Chi square test.

Three out of four (77%) students had an ambition to pursue a PhD degree (Table 1). Further to our regression models (Table 4), students were more likely to have a PhD ambition if they were satisfied with the program (as compared to those who were neutral (i.e., neither dissatisfied nor satisfied), OR 8.2, 95% CI 4.1–16.5), had a supervisor with high expectations to them (as compared to medium expectations, OR 2.1, 95% CI 1.3–3.6), or had published at least one scientific paper during their MSRP period (OR 1.8, 95% CI 1.0–3.5). That was also the case for students who were satisfied with their own efforts, as compared to those who were neutral (OR 2.7, 95% CI 1.3–5.7). On the other side, students were less likely to report a PhD ambition if they were 30 years of age or more (as compared to 20–24 years, OR 0.3, 95% CI 0.1–0.7), and reported a larger need for supervision (as compared to those who did not, OR 0.4, 95% CI 0.2–0.8)). There were no differences in gender, area of research, or evaluation period (E1 vs. E2).

**Table 4.** Students’ ambitions to pursue a PhD degree in the Norwegian medical student research program (MSRP).

	N	Odds ratio (95% CI)^a^
Gender		
Male	224	1 (ref.)
Female	182	0.7 (0.4 to 1.1)
**Age, categories**		
20–24	199	1 (ref.)
25–29	177	0.7 (0.4 to 1.2)
≥30	30	0.3 (0.1 to 0.7)
**Type of research**^b^		
Laboratory	217	1 (ref.)
Epidemiology	66	1.1 (0.6 to 2.2)
Clinical	98	1.0 (0.5 to 1.8)
Combination^c^	-	-
Other	17	1.7 (0.4 to 7.9)
**Evaluation Period**		
E1: 2002–2006	180	1 (ref.)
E2: 2007–2014	226	1.0 (0.6 to 1.7)
**Satisfaction with the MSRP**		
Dissatisfied	40	0.9 (0.4 to 2.2)
Neither dissatisfied nor satisfied	43	1 (ref.)
Satisfied	323	8.2 (4.1 to 16.5)
**Supervisors’ expectations, as perceived by students**		
Low	13	0.3 (0.1 to 1.1)
Medium	120	1 (ref.)
High	273	2.1 (1.3 to 3.6)
**Need more supervision**		
No	245	1 (ref.)
Yes	98	0.4 (0.2 to 0.8)
Do not know	63	0.5 (0.3 to 1.0)
**Publication**		
No	229	1 (ref.)
To be submitted/ under review	75	1.0 (0.6 to 2.0)
Yes	101	1.8 (1.0 to 3.5)
**Satisfaction with own effort**		
Dissatisfied	40	1 (ref.)
Neither dissatisfied nor satisfied	63	0.5 (0.2 to 1.1)
Satisfied	303	2.7 (1.3 to 5.7)

^a^Adjusted for age and gender

^b^Four possible answers; ‘public health/epidemiology’, ‘clinical/patient oriented’, ‘laboratory’, and ‘other, please specify’. The responses in the ‘other’ category were categorized as ‘combination’ if it included a combination of laboratory and either clinical or epidemiological research, and the ‘other’ category otherwise.

^c^All 8 students engaged in combination research wanted to pursue a PhD

## Discussion

In this study we have combined and compared the two first evaluations of the Norwegian MSRP and found that the high degree of satisfaction among undergraduate medical students had prevailed over time between 2002 and 2014. A clear majority were satisfied with the supervision they received. However, the second group of students (2007–14) apparently received less regular and fewer hours of supervision and reported that they needed more. Three out of four students had ambitions to pursue a PhD and a clear majority experienced that the MSRP had a positive impact on their regular medical curriculum.

The main strengths of our study is the inclusion of students from all four Norwegian Medical faculties and the high response rate at the two evaluations (88% and 86%). Both factors add to the generalizability of our findings. The use of almost identical questionnaires used in 2007 and 2015 also gave added value.

Our study also had some limitations. We did perform several comparisons in our study. Thus, we cannot exclude that some of the observed differences were due to chance. Due to the study design we only had information about the students’ ambitions to pursue a PhD, not whether they actually had completed the degree.

During the entire study period, there were no changes regarding the required academic qualifications or procedures for student admission at all four Medical faculties. However, there was a general gender shift towards more females among incoming medical students. Yet, we observed only a slight increase in the proportion of female MSRP-students between Evaluation 1 and 2 (43% vs. 48%, p = 0.38), while most other demographic characteristics were similar.

A Hawthorne (placebo) effect in terms of an initial enthusiasm may have played a role during the early years and influenced both people (academics and students) and institutions (administrators) [16]. One indication is that students were more satisfied with their supervision early on (E1) while later students clearly wanted more (E2). If that tendency persists over time, student supervision must be brought up and emphasized more clearly in daily practice as well as in further evaluations. Moreover, the importance of supervisory relationships in developing researchers, in addition to the educators’ role, must be emphasized to a greater extent, as reported by others [10,17,18].

Our findings add credibility to several published papers that encourage or report their experiences from organized – mostly intercalated – undergraduate research programs [1–6]. Others have focussed on inherent and/or organizational factors that may motivate or – mostly – limit student engagement in research while in medical schools or after graduation [4,19–23].

A majority of previous studies have emphasized the impact undergraduate research has on learning style, critical appraisal, evidence-based practice, skills acquisition, and information literacy. These are all necessary capabilities to identify and discuss ways to close knowledge gaps in medical practice [3,23–32]. Previous reports on students who are involved in undergraduate research have focused on benefits for students in terms of an improved achievement in their regular medical studies [2,6,19,20,25,27–29]. We found that almost nine out of ten (88%) students felt their research had a positive impact on their regular medical curriculum achievements. Moreover, we agree with authors who encourage students to consider the pursuit of an academic career once they graduate as medical candidates [1,2,5,15,31].

A separate survey involving a local part of our national cohort was conducted at the University of Bergen in 2017 [33–35]. As many as 14% of their admitted MSRP students had resigned from the program and for reasons that were similar to what we found. Thus, students who resigned, reported that they experienced a suboptimal relation with their supervisor, a gradual loss of interest in the project, and that they had ‘not found their place’ in the research group [34,35]. On the other hand, many previous MSRP students – even some who did not complete the program – continued to publish scientific papers years after graduation [34]. Our findings confirm that student competence, autonomy, and a good relation to the supervisor and research group are necessary elements to complete the MSRP successfully as well as for pursuing a PhD, as was also indicated by Rooij et al [11].

Further research of the current cohort will explore the career directions of the MSRP students, e.g., their pursuit of an academic track and their predicted ambitions. Later evaluations will be designed to establish if our national program continues to deliver similarly in terms of recruiting researchers to the medical profession [14,15].

In conclusion, the early favourable perceptions and satisfaction of the national MSRP prevailed over time. Recruitment of graduated physicians to medical research was maintained and both the overall satisfaction with the program and the ambition as regards the pursuit of a PhD degree was clearly met. Our data indicate that the current national program must be prolonged as a way to continue the increase in the number of physician-scientists. Nevertheless, a persistent vigilance on student satisfaction in daily scientific practice and future evaluations is clearly recommended.
